# Three-Dimensional (3D) Surface-Enhanced Raman Spectroscopy (SERS) Substrates for Sensing Low-Concentration Molecules in Solution

**DOI:** 10.3390/nano14211728

**Published:** 2024-10-29

**Authors:** Ashutosh Mukherjee, Frank Wackenhut, Alfred J. Meixner, Hermann A. Mayer, Marc Brecht

**Affiliations:** 1 Center for Process Analysis and Technology (PA&T), School of Life Sciences, Reutlingen University, Alteburgstraße 150, 72762 Reutlingen, Germany; ashutosh.mukherjee@reutlingen-university.de; 2 Reutlingen Research Institute (RRI), Reutlingen University, Alteburgstraße 150, 72762 Reutlingen, Germany; 3 Institute of Physical and Theoretical Chemistry, Eberhard Karls University of Tübingen, Auf der Morgenstelle 18, 72076 Tübingen, Germany; alfred.meixner@uni-tuebingen.de; 4 Center for Light-Matter-Interaction, Sensors and Analytics (LISA+), Eberhard Karls University of Tübingen, Auf der Morgenstelle 15, 72076 Tübingen, Germany; 5 Institute of Inorganic Chemistry, Eberhard Karls University of Tubingen, Auf der Morgenstelle 18, 72076 Tübingen, Germany; hermann.mayer@uni-tuebingen.de

**Keywords:** surface-enhanced Raman spectroscopy, silica microparticles, SERS substrates, three-dimensional SERS substrates, plasmonics, SERS in solution

## Abstract

The use of surface-enhanced Raman spectroscopy (SERS) in liquid solutions has always been challenging due to signal fluctuations, inconsistent data, and difficulties in obtaining reliable results, especially at very low analyte concentrations. In our study, we introduce a new method using a three-dimensional (3D) SERS substrate made of silica microparticles (SMPs) with attached plasmonic nanoparticles (NPs). These SMPs were placed in low-concentration analyte solutions for SERS analysis. In the first approach to perform SERS in a 3D environment, glycerin was used to immobilize the particles, which enabled high-resolution SERS imaging. Additionally, we conducted time-dependent SERS measurements in an aqueous solution, where freely suspended SMPs passed through the laser focus. In both scenarios, EFs larger than 200 were achieved, which enabled the detection of low-abundance analytes. Our study demonstrates a reliable and reproducible method for performing SERS in liquid environments, offering significant advantages for the real-time analysis of dynamic processes, sensitive detection of low-concentration molecules, and potential applications in biomolecular interaction studies, environmental monitoring, and biomedical diagnostics.

## 1. Introduction

Raman spectroscopy is highly valued for its specificity, but small Raman scattering cross-sections often limit its applicability. One approach to significantly increase sensitivity is surface-enhanced Raman scattering (SERS), where a suitable surface enhances the Raman signal [1,2]. With enhancement factors (EFs) ranging from 10^2^ to 10^8^, SERS allows for the detection of low-abundance species even in complex mixtures.

In solution, SERS enables the real-time analysis of dynamic processes and the detection of low-concentration molecules with good sensitivity and specificity [3,4,5,6,7,8]. However, SERS in solution poses several challenges due to its three-dimensional (3D) geometry. The enhancement of the Raman signal in conventional two-dimensional (2D) SERS substrates is limited to a few tens of nanometers above the surface [9,10,11]. Dynamic and complex solutions, particularly biological fluids and environmental samples, require SERS substrates to perform reliably in these 3D environments. Hence, there is a growing need for effective SERS platforms that operate in solution [12,13].

Silica microspheres functionalized with silver (Ag) nanoparticles (SMP@Ag) have emerged as promising candidates for such applications. These substrates combine the high surface area and ease of modification of silica microspheres with the plasmonic properties of Ag NPs, offering an effective 3D SERS platform [14,15]. The fabrication and characterization of mercapto-functionalized silica microspheres (SMPs), subsequently decorated with silver (Ag) nanoparticles using Tollens’ reagent (SMP@Ag), has already been described [15]. Three-dimensional (3D) substrates demonstrate excellent performance in complex matrices and have the potential for multiplexed detection, thus broadening their application in diagnostics, environmental monitoring, and chemical sensing. Additionally, they can be suspended in a solution or any biological environment and distributed across different spatial locations.

In comparison to similar SERS platforms reported in the literature, a few studies have employed different strategies to achieve SERS in solution or on dried samples, resulting in notable variations in EFs, sensitivity, and detection limits. For instance, Li et al. developed poly(styrene-co-acrylic acid) core-silver nanoparticle/silica shell microspheres, demonstrating high sensitivity [16,17]. In contrast, Liu et al. presented SiO_2_@Ag core–shell particles that exhibited strong SERS enhancement [18]. Similarly, Jensen et al. focused on single-molecule detection using highly sensitive pH-responsive substrates, achieving single-molecule sensitivity [19]. Kim et al. described a CRISPR-mediated SERS assay that achieved remarkable femtomolar sensitivity for bacterial DNA detection [20]. Chen et al. investigated silver nanoparticle-decorated carbon nanospheres for melamine detection, demonstrating strong SERS activity in solution [21]. Cai et al. developed gold-coated sulfonated polystyrene microspheres, enabling nanomolar sensitivity for 4-aminothiophenol (4-ATP) [22]. Trinh et al. uniquely combined solar-to-steam generation with SERS detection, achieving nanomolar sensitivity without quantifying the EF, offering a dual-functional platform that sets it apart from traditional SERS substrates [23]. Lastly, Lupa et al. reported relatively low EFs (ranging from 2.75 to 5.75) for Au NPs-coated SMPs [24]. This diversity in substrates emphasizes the difficulties in directly comparing EFs and detection capabilities due to differing methodologies and measurement conditions, especially when contrasting solution-based and dried-sample environments.

This study investigates two distinct scenarios for 3D-SERS, each contributing novel insights to the field.

In the first, stationary scenario, SMP@Ag particles were dispersed in a highly viscous solution of methylene blue (MB) and glycerin. In this case, glycerin served as a medium to immobilize the SMP@Ag particles. This use of glycerin enabled high-resolution SERS imaging within a 3D matrix, allowing for the acquisition of spatially resolved SERS spectra of analytes across the substrate. This technique provides a unique method for mapping analyte distribution in solution, thereby enhancing the utility of SERS substrates in complex environments.

In the second scenario, referred to as the dynamic scenario, SMP@Ag particles were freely floating in a water–MB solution, simulating real-world conditions where substrates were suspended in fluidic environments. Here, we introduced time-dependent SERS measurements to capture the temporal fluctuations in the SERS signal as the particles moved through the laser focus. This capability enables the real-time detection of low-concentration analytes, which is essential for continuous monitoring in environments such as biological fluids and environmental samples. Additionally, we employed mercapto-functionalized silica spheres for immobilizing metal NPs, marking a novel fabrication strategy. Combined with the straightforward fabrication of these SMP@Ag substrates using Tollens’ reagent, this study presents a versatile and innovative approach to the development of 3D SERS substrates.

This method’s excellent, reproducible, and reliable performance, coupled with its simple fabrication, means it can be produced at a low cost. It is helpful for real-time, on-site SERS detection in fields like analytical chemistry, biomedical research, environmental research, and forensic science. Three-dimensional (3D) SERS substrates provide valuable depth information, advancing SERS-based sensing and enabling new scientific insights.

## 2. Materials and Methods

### 2.1. Fabrication of Mercapto-Functionalized SMPs

The functionalized SMPs were prepared according to a previously published protocol regarding silica particles with similar morphology but different functionalization [25,26]. A solution containing 115 mL of water and 0.266 mL of aqueous ammonium hydroxide (NH_4_OH) was stirred continuously for 40 min. A syringe pump added 2.6 mL of a mixture of tetraethoxysilane (TEOS) and mercapto-trimethoxysilane (MerTMS) with a ratio of 22 mmol:88.3 mmol. The amount and composition of this mixture allowed for the control of the particles’ shape, size, and morphology. After adding the silane mixture, the solution was stirred at room temperature for 14 h. The solvent was removed by washing with water and ethanol, and the SMPs were dried in an oven at 70 °C for 16 h. The resultant SMPs were non-porous, monodispersed, approx. 3 µm sized mercapto-functionalized spherical silica particles. The details can be found in reference [15].

### 2.2. Synthesizing SMPs with Metal Nanoparticles

#### Tollens’ Reagent (Ag)

Tollens’ reagent was used to precipitate elemental Ag, producing a Ag mirror on the surface of the SMP. For this, Ag nitrate (AgNO_3_) 53.0 mg—312 μmol, aqueous ammonia solution (NH_3_) 25%—380 μL, and saturated aqueous D-(−)-Fructose solution (2.0 mL) were dissolved in 3 mL of deionized water. Subsequently, one small pellet of NaOH was added and stirred until visibly dissolved. A total of 500 µL of this resulting solution was added to an Eppendorf tube containing mercapto-functionalized SMPs and was stirred continuously to allow Tollens’ reagent to react uniformly with the complete surface area, generating SMP@Ag particles. Then, the mixture in the Eppendorf tube was allowed to rest for 1 min at room temperature. Afterward, the Eppendorf tube was placed in an ice bath to stop the chemical reaction, and the contents were washed with deionized water.

### 2.3. SERS Experiments

A commercial microscope from WITec (Oxford Instruments, Ulm, Germany) alpha300RAandS was used for the SERS experiments. A 532 nm diode laser with a nominal output power of 40 mW was used for excitation. The illumination and collection of the detected signal were carried out using two configurations illustrated in Supplementary Materials in Figure S1. Figure S1a illustrates the top excitation/detection configuration using an objective lens (Carl Zeiss, Jena, Germany; EC Epiplan, 20×, NA = 0.4). This configuration was suitable for dynamic experiments, as the 3D SERS substrates were suspended in the solution. Figure S2b shows bottom excitation/detection by an objective lens (NIKON 60×, NA = 0.55, Tokyo, Japan). In static/stationary experiments, high-resolution spatial images of the substrate within the solution were possible due to the nature of the solution. To achieve this, a bottom illumination/detection configuration was used. Multi-mode fibers with different diameters guide the detected signal to the spectrometer. The diameter (10 µm, 25 µm, 50 µm, and 100 µm, all with an NA = 0.12) of these fibers determines the confocal pinhole size [27]. For detection, the microscope is equipped with a lens-based ultra-high-throughput spectrometer (UHTS 300) with a thermoelectrically cooled (down to −60 °C) back-illuminated CCD and an EMCCD (Andor DU970N-BV, Belfast, UK). All SERS experiments were performed using a 600 L/mm grating, which offers an extensive spectral range. Data processing for all the above measurements was performed using Control 5.0 software provided by WITec (Ulm, Germany). All experiments were carried out under ambient conditions.

### 2.4. Preparation of Solution

SERS experiments were performed with methylene blue (MB) as an analyte (purchased from Carl Roth Gmbh & Co. Kg, Karlsruhe, Germany). MB exhibits an absorption peak at 665 nm, a smaller peak at 610 nm, and an additional peak at 293 nm [28].

#### 2.4.1. Glycerin–MB Solution

Glycerin (99% pure) was put into a glass beaker, and a few milligrams of MB was added to achieve a concentration of MB of 3 × 10^−5^ M. After adding MB, the solution was vigorously stirred for an extended period inside the glass beaker using a spatula. The SMP@Ags were then added to this mixture and blended well. The mixture was sonicated for 5 min to prevent the aggregation of the SMP@Ag particles inside the solution. With significantly extended sonication times, there is a chance that Ag NPs might separate from the SMP@Ags.

#### 2.4.2. Water–MB Solution

Distilled water was poured into a beaker with a few milligrams of MB to obtain an MB concentration of 3 × 10^−5^ M. The solution was mixed briefly before being placed in a small ceramic Petri dish to be tested for SERS. After that, SMPs treated with Tollens’ reagent were added to the solution and left to float for a while before the SERS measurements.

### 2.5. Scanning Electron Microscopy (SEM)

SEM images were acquired with a HITACHI SU8030 (Tokyo, Japan) at 2 kV, a secondary electron detector, and an accelerating voltage of 0.5 to 30 kV.

A detailed characterization of such SMP@Ags using SEM, energy dispersive X-ray (EDX), and dark field scattering are shown in our previous publication [15].

## 3. Results and Discussion

The preparation of SMPs functionalized with Ag nanoparticles (SMP@Ag) was already discussed in a previous publication. The SMP@Ags were characterized by scanning electron microscopy (SEM), energy dispersive X-ray (EDX), and dark-field scattering, and their SERS activity was investigated in a stationary 2D environment [15]. These particles provide multiple resonances throughout the visible and near-infrared spectral region within the same SMP, thus enabling multi-analyte screening [9,15].

**Figure 1:**
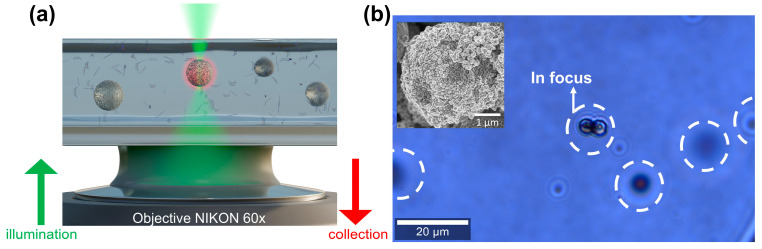
(**a**) Schematics (not to scale) of bottom illumination/collection configuration for SMPs@Ags immersed in a solution of glycerin and MB, and (**b**) microscopic (bright-field) image of SMP@Ags mixed in glycerin–MB. The image shows that the SMP@Ags are localized in different planes in the solution. Inset: SEM image of an SMP@Ag.

**Figure 2:**
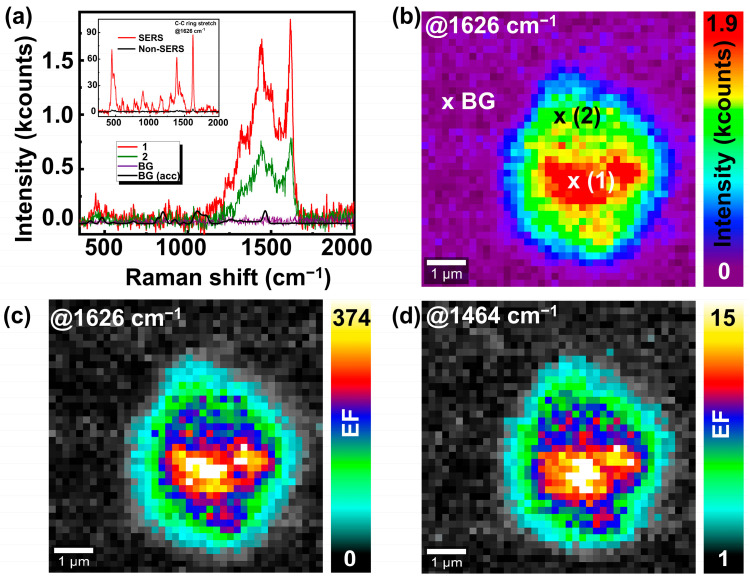
(**a**) SERS spectra of glycerin and MB at different spatial positions on SMP@Ags, position 1 (bright region), position 2 (low-intensity region), background (BG), and BG accumulated (BG acc), acquired separately from the same solution with 500 accumulations. Inset: SERS spectra and non-SERS spectra of MB in water (measured separately only for reference). (**b**) SERS image of an SMP@Ag immersed in glycerin–MB solution at 1626 cm^−1^. The marks show the positions where the spectra in (**a**) are acquired. (**c**) Spatial distribution of EF for the MB peak at 1626 cm^−1^ and (**d**) spatial distribution of EF for the glycerin peak at 1464 cm^−1^.

The SMP@Ags were immobilized by glycerin for the first experiments. The immobilization enables the acquisition of SERS spectra at specific points on the SMP@Ag particle and SERS imaging. Apart from MB, glycerin serves as a second analyte. A sizable drop of the glycerin–MB solution containing SMP@Ags was deposited onto a glass slide, according to the scheme in Figure 1a. A microscopic image of the SMP@Ags immersed in this solution is shown in Figure 1b. The SMP@Ags in the center of Figure 1b are within the microscope’s focal plane, while the others are out of focus. The SEM picture in the inset of Figure 1b depicts the morphology of the SMP@Ags and shows Ag particles with sizes ranging from 10 nm to 400 nm on the SMP surface. Due to the high viscosity of glycerin, the position of the SMP@Ags remains stationary within the timescale of the subsequent experiments, enabling extended optical measurements. Raman imaging in a bottom excitation/detection configuration was optimal for conducting SERS experiments and high-resolution imaging within the solution; the results are shown in Figure 2.

Figure 2a depicts the SERS spectra acquired at the different spatial positions on a single SMP@Ag (as marked in Figure 2b) in a glycerin–MB solution. The spectrum at position 1 (red in Figure 2a) is obtained from a bright region on the SMP@Ag particle, and the green spectrum in Figure 2a is acquired at position 2 with lower intensity. The background (BG, purple in Figure 2a) spectrum is recorded beside the SMP@Ag. The SERS spectrum of glycerin–MB deviates significantly from the MB spectra (shown in the inset). This can be ascribed to the additional presence of glycerin. For example, the most intense peak of MB is at 1626 cm^−1^, corresponding with the C-C ring stretch [28], while the signal at 1464 cm^−1^ is due to the CH_2_ vibration of glycerin [29]. No Raman signal from MB or glycerin can be observed in the BG spectrum in Figure 2a. Hence, it was necessary to significantly increase the acquisition time to obtain any observable signal from the BG. The black spectrum shows the accumulated BG spectrum (BG acc) for 500 accumulations. Without SERS enhancement, glycerin dominates the spectrum, and only a weak MB Raman signal is visible in the close-up view of the spectrum, as shown in the Supplementary Materials in Figure S2. The SERS image in Figure 2b was based on the integrated intensity of the most intense MB peak (1626 cm^−1^) with a spectral width of 20 cm^−1^. The small region shown in red exhibits the strongest SERS enhancement. Additional SERS images of single SMP@Ags immersed in a solution of glycerin–MB are presented in Figure S3 of the Supplementary Materials. The EF was determined using the Raman signals in the accumulated BG (BG acc) spectrum. The EF is calculated as the intensity ratio of the most intense peak of MB (at 1626 cm^−1^) on the SMP and the substrate:

(1) $$EF=\frac{I_{Hotspot}}{I_{Substrate}}$$

$I_{Hotspot}$ is the intensity of a specific Raman peak at the bright regions and $I_{Substrate}$ is the intensity of the same peak on the substrate (glass slide) without any metal NPs. The reason behind deliberately employing this method to calculate the SERS EF stems from the inherent difficulties to accurately estimate the number of molecules present within a given spot, their exact location in the hot spot, and their distance from the surface [30,31,32]. It is important to note that the method used to calculate EF in this study results in lower EF values compared to those reported in other SERS research, which typically range from 10^2^ to 10^10^ [5,33,34]. Figure 2c,d show the spatial distribution of EFs at the characteristic Raman frequencies of MB (at 1626 cm^−1^) and glycerin (at 1464 cm^−1^), respectively, derived from the identical dataset as presented in Figure 2b. The spatial EF distributions for MB and glycerin are quite similar. Strong differences are observed for the maximum EFs of glycerin (EF_max_ = 15) and MB (EF_max_ = 374). This could be due to MB accumulating on the SMP@Ag due to the electrochemical properties of MB containing a sulfur atom within its structure. Additionally, the large surface area of SMP@Ag provides numerous adsorption sites, facilitating the accumulation of MB molecules. This combined effect leads to a higher effective concentration of MB on SMP@Ags, contributing to the enhanced signal and larger EF of MB compared to glycerin. The maximum (EF_max_) and minimum (EF_min_) EF calculations for Figure 2c,d are shown in Supplementary Materials.

**Figure 3:**
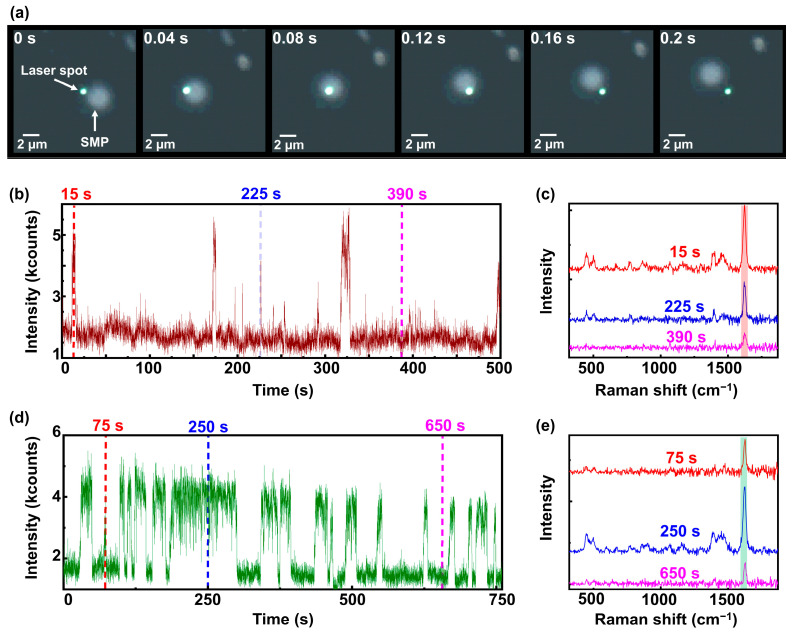
(**a**) Video snapshots of SMP@Ags immersed in a water–MB solution passing through a laser spot at different time frames. (**b**) SERS intensity of MB (at 1626 cm^−1^) measured as a function of time (for a preset duration of 500 s) for SMP@Ags passing through the laser focus spot in a water–MB solution. (**c**) SERS spectra of MB for the corresponding time frames shown in (**b**). (**d**) SERS intensity of MB (at 1626 cm^−1^) acquired after a resting time, also measured as a function of time (for a preset duration of 750 s) for SMP@Ags passing through the laser focus spot in a water–MB solution. (**e**) SERS spectra of MB for the corresponding time frames shown in (**d**).

The results shown in Figure 2 were obtained for immobilized SMP@Ags. However, in less viscous solutions, the SMP@Ags will not remain stationary. Consequently, freely floating SMP@Ags are investigated in a water–MB solution. The results are shown in Figure 3.

Next, the laser was focused in the water–MB solution in a beaker, and the detection was in a top excitation/detection configuration (back-scattering detection geometry) for easier alignment with the sample and to ensure efficient capture of scattered light from the solution. Several videos were recorded in the bright-field mode of the microscope, showing the movement of SMP@Ags in solution. Figure 3a presents snapshots from one of these videos, illustrating an SMP@Ag (located out of the focal plane) moving through a much smaller laser spot (approx. 700 nm) than the size of the particles (approx. 3 µm). The images suggest that the laser focus widens as the SMP@Ag passes through it. This is attributed to the increased signal intensity resulting from the scattering of the Ag nanoparticles on the SMP, which in turn causes the camera to reach saturation.

Additionally, a SERS spectrum was acquired every second with an integration time of 500 ms. Figure 3b displays the temporally resolved SERS intensity of MB at 1626 cm^−1^. In Figure 3b, intensity spikes are observable, for instance, around the 15 s mark, indicating the passage of SMP@Ags through the laser focus volume, resulting in a noticeable enhancement of the MB SERS signal. At approximately 175 s and 315 s, extended periods with high SERS intensity suggest a passage of multiple SMP@Ags or a single slowly moving SMP@Ag. However, there are several intensity spikes with durations shorter than 3 s, e.g., at 200 s, 210 s, and 225 s, where a single SMP@Ag swiftly passes the excitation focus.

Figure 3c shows Raman spectra at three distinct time points, i.e., 15 s, 225 s, and 390 s, indicated by the dashed lines in Figure 3b. The spectrum at 390 s resembles the BG level. Here, only the most intense MB peak at 1626 cm^−1^ is visible. The spectrum at 225 s shows a clear MB SERS signal when the SMP passes the laser focus. However, the enhancement strongly depends on the actual position of the SMP@Ag to the excitation focus, the number of Ag nanoparticles on the SMP, and their distribution on the surface, which can vary between different SMP@Ags [15].

After a resting period of 10 min, the experiment was repeated, as shown in Figure 3d. At first glance, the dwell time of SMP@Ags in the laser focus is increased compared to Figure 3b. In Figure 3d, high-intensity periods are observed for extended periods of several seconds, and only one very short intensity increase is visible at around 75 s. Figure 3e shows Raman spectra at three distinct time points, i.e., 75 s, 250 s, and 650 s, indicated by the dashed lines in Figure 3d. The spectrum at 650 s resembles the BG level. Here, only the most intense MB peak at 1626 cm^−1^ is visible. The spectrum at 250 s shows a clear MB SERS signal when the SMP passes the laser focus. This observation suggests that the SMP@Ag movement is slowing down or that the SMP@Ags have clustered. However, this situation is advantageous for sensing applications due to the prolonged high-intensity phases.

**Figure 4:**
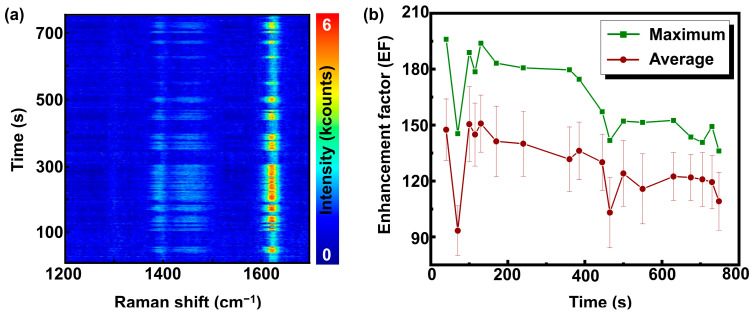
(**a**) Surface plot of MB spectra spanning 750 s ranging from 1200 cm^−1^ to 1700 cm^−1^ and (**b**) maximum and average EFs for the SMPs in water–MB solution.

Figure 4a shows a 2D surface plot of the Raman spectra (shown in Figure 3d,e) in the range from 1200 cm^−1^ to 1700 cm^−1^. The simultaneous fluctuation of all significant lines indicates an enhancement of all Raman lines of MB. In the next step, the time-dependent EF is calculated. First, the dataset is subdivided into 18 high-intensity periods, and then the maximum and average EF is calculated for each period according to Equation (1). A non-SERS spectrum was acquired separately from a solution of the same concentration and under the same experimental conditions without the addition of SMP@Ags.

The result is given in Figure 4b. The maximum EF using Equation (1) is calculated using the maximum intensity within a certain high-intensity period, whereas the average EF is based on the average intensity over the whole period. The maximum and average EF vary depending on the precise location of the SMP@Ag in the laser focus. The largest maximum EF observed in this dataset is approx. 200, whereas the average EF can reach up to 150 ± 20. As previously mentioned, the EF values obtained in this study are lower due to the specific method of calculation employed, as outlined in Equation (1). The EF for the water–MB solution is lower than the glycerin–MB solution, likely due to the rapid movement of SMP@Ag particles through the laser focus in the water–MB experiments. This reduces measurement time, which may be less than intended. Additionally, in the water–MB solution, SMP@Ag particles constantly move across different planes, leading to instances where particles detected at the laser focus may not be fully in focus. These factors contribute to the differences in EF observed between the glycerin–MB and water–MB solutions. The maximum and average EF graphs exhibit a declining trend; this might result from gradual changes in the focus position in the solution.

## 4. Conclusions

This study examined SMPs functionalized with Ag nanoparticles in two different 3D environments: a stationary and a dynamic scenario.

In the stationary scenario, glycerin was used to immobilize the SMP@Ags, making SERS imaging within the solution possible. Furthermore, this scenario mimics a situation where the SMP@Ags are immobilized in, e.g., specific cell compartments or tissue. Along with MB, glycerin can function as an analyte to further investigate the use of enhancing multiple analytes within the same sample. Glycerin and MB both showed good enhancement, although their EFs differed significantly. The EF can reach up to 374 for MB, whereas for glycerin, it can reach up to 15. This indicates that multiple analytes are enhanced on the same SMP.

In the dynamic scenario, MB solution in water was used. Here, the SMP@Ags were mobile and randomly floating. Time-dependent experiments were performed, where the MB’s SERS signal was detected when an SMP@Ag passed through the laser focus. An SMP@Ag traveled across the laser EF in video snapshots at different time frames. The results indicate that single and clustered SMP@Ags pass the laser focus. SERS enhancement can be achieved with the SMPs functionalized with Ag nanoparticles and EFs up to 200.

The novel use of mercapto-functionalized silica spheres as a natural ‘glue’ for immobilizing metal NPs in 3D SERS substrates, along with the easy fabrication using Tollens’ reagent, represents an important advancement. Furthermore, the glycerin-immobilized system allowed for detailed spatial SERS imaging within a 3D matrix, while the dynamic system enabled real-time detection of low-concentration analytes in solution. In the long term, these findings could pave the way for innovative applications in biomedicine and environmental science, particularly in developing more effective 3D SERS substrates. These might detect and identify a broader range of analytes in complex 3D environments, such as cells, tissues, pollutants, and trace elements in water or other solutions.

## Data Availability

The datasets generated and analyzed during the current study are not publicly available since they are part of an ongoing PhD thesis. However, they are available from the corresponding authors upon reasonable request.

## Supplementary Materials

The following supporting information can be downloaded at https://www.mdpi.com/article/10.3390/nano14211728/s1, Figure S1: Schematics of experimental details that illustrate top and bottom excitation and detection configurations of WITec alpha 300RA&S Raman microscope; Figure S2: Raman spectrum of glycerin–MB solution (with 500 accumulations) with most intense peaks marked; Figure S3: Additional examples of SERS imaging of SMP@Ags in glycerin–MB solution.
