# Supplementary material for: Three-Dimensional (3D) Surface-Enhanced Raman Spectroscopy (SERS) Substrates for Sensing Low-Concentration Molecules in Solution
Source: Nanomaterials (Basel). 2024 Oct 29;14(21):1728. doi: 10.3390/nano14211728 (PMC11547942; doi:10.3390/nano14211728)
Supplement: Supplementary file 1 [file nanomaterials-14-01728-s001.zip › nanomaterials-3260244-supplementary.pdf]

Supplementary Materials

Three-dimensional (3D)-Surface-Enhanced Raman Spectroscopy (SERS) Substrates for Sensing Low Concentration Molecules in Solution

Ashutosh Mukherjee [1,2,3], Frank Wackenhut [1,2,*], Alfred J. Meixner [3,4], Hermann A. Mayer [5] and Marc Brecht [1,2,3,4,*]

[1] Center for Process Analysis and Technology (PA&T), School of Life Sciences, Reutlingen University, 72762 Reutlingen, Germany; ashutosh.mukherjee@reutlingen-university.de
[2] Reutlingen Research Institute (RRI), Reutlingen University, 72760 Reutlingen, Germany;
[3] Institute of Physical and Theoretical Chemistry, Eberhard Karls University of Tübingen, 72074 Tübingen, Germany; alfred.meixner@uni-tuebingen.de
[4] Center for Light-Matter-Interaction, Sensors and Analytics (LISA+), University of Tübingen, 72074 Tübingen, Germany
[5] Institute of Inorganic Chemistry, Eberhard Karls University of Tübingen, 72074 Tübingen, Germany; hermann.mayer@uni-tuebingen.de
* Correspondence: Correspondence: frank.wackenhut@reutlingen-university.de (F.W.); marc.brecht@reutlingen-university.de (M.B.)

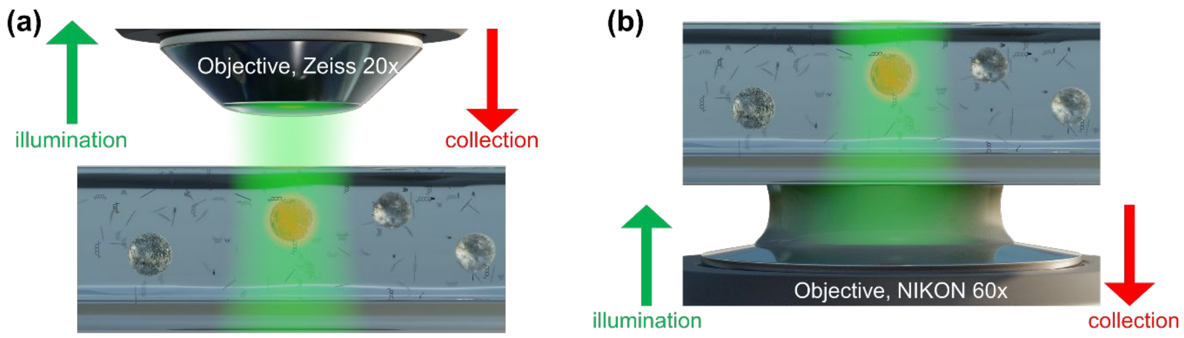

Figure S1. (a) Schematics of top-excitation and detection configuration through an objective lens (Carl Zeiss; EC Epiplan, 20×, NA = 0.4) at WITec alpha300RA&S, and (b) Schematics of bottom-excitation and detection configuration through an objective lens (NIKON 60×, NA = 0.55) at WITec alpha300RA&S.

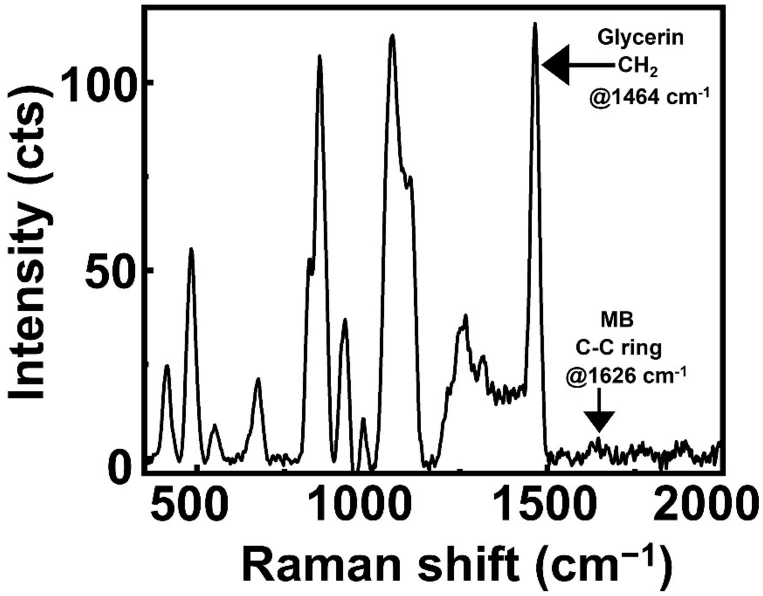

Figure S2. Raman spectrum of glycerin -MB solution marked with their most intense peaks.

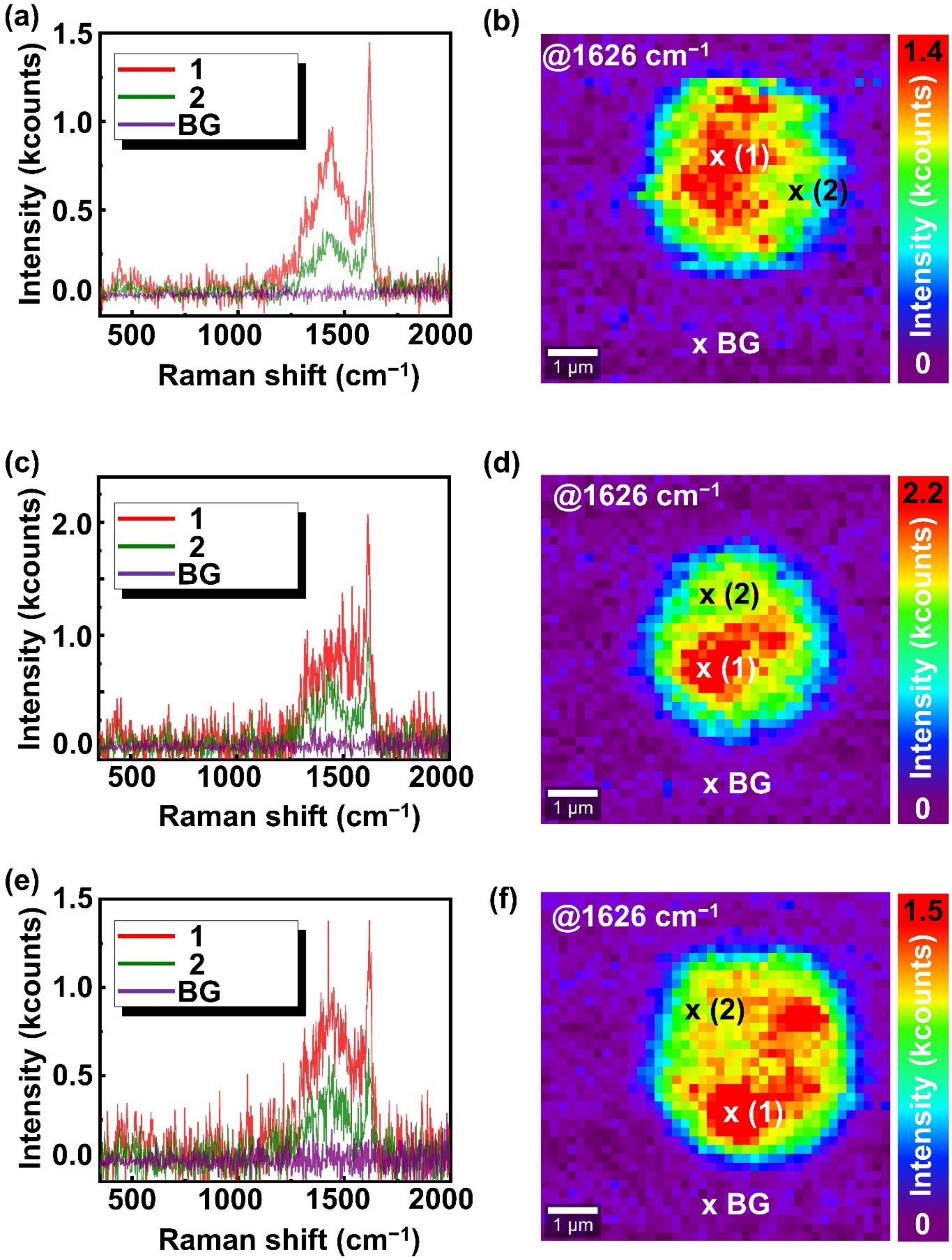

Figure S3. Example 1 - (a) SERS spectra of glycerin and MB at different spatial positions on SMP, position 1 (bright region), position 2 (low-intensity region), background (BG), (b) SERS image of a SMP immersed in glycerin-MB solution at 1626 cm^{-1}. The marks show the positions where the spectra in (a) are acquired, Example 2 - (c) SERS spectra of glycerin and MB at different spatial positions on SMP, position 1 (bright region), position 2 (low-intensity region), background (BG), (d) SERS image of a SMP immersed in glycerin-MB solution at 1626 cm^{-1}. The marks show the positions where the spectra in (c) are acquired, and Example 3 - (e) SERS spectra of glycerin and MB at different spatial positions on SMP, position 1 (bright region), position 2 (low-intensity region), background (BG), (e) SERS image of a SMP immersed in glycerin-MB solution at 1626 cm^{-1}. The marks show the positions where the spectra in (e) are acquired.

EF calculations

The calculated enhancement factor (EF) for Figures 2c and 2d are as follows:

Please note that the same data set shown in Figure 2b was used to calculate the EF in Figures 2c and 2d. Since the background (BG (acc)) was used to calculate the EF:

For methylene blue (MB):

$$EF\ (for\ MB) = \frac{I_{Hotspot}}{I_{Substrate}} = \frac{I_{Hotspot\ MB}}{I_{BG\ (acc)}} = \frac{1900}{5} = 374$$

Therefore, 374 is the highest value on the z-color scale bar of Figure 2c, which has been corrected in the main manuscript.

For the lowest EF value on the z-color scale of Figure 2c:

Since the intensity of MB is obscured by noise and not visible, the intensity is considered to be 0

$$EF\ (for\ MB) = \frac{I_{Substrate}}{I_{Substrate}} = \frac{I_{Substrate\ MB}}{I_{BG\ (acc)}} = \frac{0}{5} = 0$$

Thus, 0 is the lowest value on Figure 2c's z-color scale. The manuscript has been updated accordingly.

Similarly, for glycerin:

$$EF\ (for\ Glycerin) = \frac{I_{Hotspot}}{I_{Substrate}} = \frac{I_{Hotspot\ Glycerin}}{I_{BG\ (acc)}} = \frac{1700}{115} = 14.79 \approx 15$$

Hence, 15 is the highest value on the z-color scale bar of Figure 2d.

For the substrate:

$$EF\ (for\ Glycerin) = \frac{I_{Substrate}}{I_{Substrate}} = \frac{I_{Substrate\ Glycerin}}{I_{BG\ (acc)}} = \frac{85}{115} = 0.73 \approx 1$$
